# The acyl-CoA Synthetase, *pudgy*, Promotes Sleep and Is Required for the Homeostatic Response to Sleep Deprivation

**DOI:** 10.3389/fendo.2018.00464

**Published:** 2018-08-21

**Authors:** Matthew S. Thimgan, Natalie Kress, Josh Lisse, Courtney Fiebelman, Thomas Hilderbrand

**Affiliations:** ^1^Department of Biological Sciences, Missouri University of Science and Technology, Rolla, MO, United States; ^2^Department of Neuroscience, Washington University School of Medicine in St. Louis, St. Louis, MO, United States

**Keywords:** acyl-CoA synthetase, sleep deprivation, sleep regulation, lipid metabolism, sleep fragmentation, Drosophila, lifespan

## Abstract

The regulation of sleep and the response to sleep deprivation rely on multiple biochemical pathways. A critical connection is the link between sleep and metabolism. Metabolic changes can disrupt sleep, and conversely decreased sleep can alter the metabolic environment. There is building evidence that lipid metabolism, in particular, is a critical part of mounting the homeostatic response to sleep deprivation. We have evaluated an acyl-CoA synthetase, *pudgy* (*pdgy*), for its role in sleep and response to sleep deprivation. When *pdgy* transcript levels are decreased through transposable element disruption of the gene, mutant flies showed lower total sleep times and increased sleep fragmentation at night compared to genetic controls. Consistent with disrupted sleep, mutant flies had a decreased lifespan compared to controls. *pdgy* disrupted fatty acid handling as *pdgy* mutants showed increased sensitivity to starvation and exhibited lower fat stores. Moreover, the response to sleep deprivation is reduced when compared to a control flies. When we decreased the transcript levels for *pdgy* using RNAi, the response to sleep deprivation was decreased compared to background controls. In addition, when the *pdgy* transcription is rescued throughout the fly, the response to sleep deprivation is restored. These data demonstrate that the regulation and function of acyl-CoA synthetase plays a critical role in regulating sleep and the response to sleep deprivation. Endocrine and metabolic signals that alter transcript levels of *pdgy* impact sleep regulation or interfere with the homeostatic response to sleep deprivation.

## Introduction

There is an increasing recognition that lipid metabolism contributes to sleep and wakefulness regulation and the response to sleep deprivation. The involvement of lipids supports the energy hypothesis of sleep regulation, in which energy is required to perform the restorative actions of sleep as well as to carry out waking activities (1). In addition, mobilized lipids can act as signaling molecules and change the composition of membranes within the cell (2). Mutations that alter free fatty acid mobilization reduce sleep rebound and protect the animal from the cognitive deficits usually seen with sleep deprivation (3). In addition, knockdown of two presumptive acyl-CoA synthetases reduces the homeostatic response to sleep deprivation and had metabolic phenotypes (4). Mutations in fatty acid binding protein result in reductions of sleep in flies and mammals (5, 6). In mammals, TRIB1 has been linked with population differences in sleep regulation (7) and mutations in fatty acid metabolism alters REM sleep (8). Plasma levels of lipid-related molecules are altered through sleep deprivation (9), and free fatty acids are elevated during sleep restriction (10). Lipid metabolism genes are found as genes that are the most changed in response to sleep deprivation in unbiased array or network analyses (11, 12). Thus lipid metabolism, and in particular free fatty acid handling plays an important, yet ill-defined, role in sleep regulation.

Acyl-CoA synthetases (ACSs) are enzymes that attach a CoA moiety to fatty acids to activate them. The resulting acyl-CoA molecule is an “activated” fatty acid that is retained in the cell, and the acyl-CoA can go on to fulfill energy needs, carry out signaling properties, or other functions within the cell or in other cells (2). Each organism has multiple ACSs with specific organ and cellular expression patterns (13). ACSs also have substrate specificity, in which different chain length fatty acids are conjugated to CoA by different ACSs (13). Knockout and knockdown data from mice reveal increases and decreases in lipid storage likely resulting from defects in the dynamics of lipid mobilization (14–16). In flies, mutations in the ACS, *bubblegum* (*bgm*), result in a severe neurodegeneration phenotype that can be rescued by fatty acid consumption (17). In flies, knockdown of two ACSs reduces the homeostatic response to sleep deprivation (4). Thus, ACSs play crucial role in energy and metabolic management and are responsive to changing physiologic conditions.

The ACS *pudgy* (*pdgy*) is upregulated under starvation conditions to continue to fulfill energy needs in the face of decreased energy consumption (18). Transcript levels of pdgy also vary between sleep and wake states in the fly (11). In this manuscript, we used a behavioral genetics approach to assess whether *pdgy* impacts the response to sleep deprivation and whether there were accompanying metabolic changes. We found that decreasing the expression of *pdgy* eliminated the homeostatic response to sleep deprivation. In contrast to the other ACSs observed before, *pdgy*, reduced baseline sleep and increased the fragmentation of the animal. Rescue of *pdgy* in a mutant background restored the sleep phenotypes. These results suggest that *pdgy* regulates sleep through the activation of fatty acids. Given that endocrine factors can alter the transcript levels of ACSs, our data suggest a mechanism by which endocrine factors can alter sleep regulation and lipid mobilization in response to sleep deprivation.

## Materials and methods

### Flies and husbandry

Flies were reared in standard laboratory conditions, such light:dark schedule, standard food (yeast, sucrose, corn syrup, molasses, and agar), 25°C and 50% humidity. The *UAS-Pdgy*^*RNAi*^ line was obtained from the Vienna *Drosophila* Resource Center (19). *Actin-GAL4*/*CyO* (*Act-GAL4*), *pdgy*^*BG*02662^, *pdgy*^*EY*02124^ (20), *Mi{PT-GFSTF.0}pdgy*^*MI*04730−*GFSTF*.0^, and flies to mobilize the transposon were obtained from the Bloomington Stock Center (Bloomington, Indiana). *pdgy*^*BG*02662^ has a 8,447 bp transposon inserted in the 5′ untranslated region and *pdgy*^*EY*02124^ has a 10,908 bp transposable element within the coding region of the *pdgy* gene (21). Background controls were generated by mobilizing the P-element using the Δ*2-3* version of the transposase to extract the transposable element, termed “revertants.” The *Mi{PT-GFSTF.0}pdgy*^*MI*04730−*GFSTF*.0^ uses a Minos transposable element that integrates into an intronic region with a splice acceptor such that the GFP tag is expressed in the protein under the control of endogenous enhancers and repressors (22). The *Fat body-GAL4* (*FB-GAL4*) was generously provided by Ronald Kuhnlein and the *UAS-pdgy*^*wt*^ was generously provided by Aurelio Teleman.

### Sleep measurements, sleep deprivation, and starvation

Sleep in flies was measured using the Trikinetics *Drosophila* Activity Monitor (DAM) system. Activity counts were converted into sleep using the protocol developed previously (23) in which data was collected in 1 min bins and 5 min of inactivity as the empirically-derived definition of sleep (23, 24). Sleep architecture and sleep metrics were calculated using an in-house program (23–25).

Sleep deprivation was accomplished using the geotaxis method as described in (3). Briefly, 3-5 day old female flies were loaded into 65 mm tubes with food on one end and an air permeable plug on the other end. After 2 full days of baseline, flies were sleep deprived using the SNAP device. Flies were deprived of sleep for 12 h between ZT12 (lights out) to ZT0 (lights on) at which point flies were released into recovery where they remained unperturbed for 48 h. Increases in sleep were calculated from the second day of baseline and presented as a percentage of sleep lost.

Starvation experiments were carried out similarly, in which flies were allowed 2 days of baseline sleep on normal food and then put onto 1% agar and water during the starvation period. For starvation tolerance assays, flies were loaded into the DAMS system for continuous monitoring and which provided activity resolution to the nearest hour to identify when the fly died.

### Real-time polymerase chain reaction (RT-PCR)

Total RNA was isolated from 20 fly heads using Trizol (Invitrogen, Carlsbad, CA) and cleaned of contaminating DNA using DNAse I. cDNA synthesis was performed in quadruplicate using Superscript (Invitrogen, Carlsbad, CA), according to manufacturer protocol. Equal amounts of cDNA were used as a starting material to amplify *RP49*. cDNA from reverse transcription reactions with comparable RP49 levels were compared. Expression values for *RP49* were used to normalize results between groups. For flies maintained on an LD schedule, both experimental and untreated controls, were collected at the same circadian time, ZT0-1 for sleep deprived or starved animals. Relative levels of transcript were determined using the ΔΔ method as described (26) to determine the relative levels of transcript between genotype or experimental flies.

### Longevity assay

Three-day-old flies were randomly assigned to one of 3 vials of 10 flies. Flies were counted and transferred onto new food every 3 times per week. Flies alive were expressed as a percentage of the original starting number of flies. Lifespan curves were analyzed using Kaplan-Meier analysis to determine significant differences.

For peroxide tolerance, individual flies were loaded into the DAMS system. After 2 days on normal food, the flies were put onto normal food containing 1% hydrogen peroxide (27) and monitored continuously until the animal died. Deaths were visually confirmed.

### Triglyceride measurements

For each genotype, 10 female flies were frozen and stored at −80°C. Lipid measurements were carried similar to (3). Briefly, flies were weighed and homogenized in a 2:1 (methanol:chloroform) solution to extract the lipids (28). The MeOH:chloroform is evaporated using the speed vac, and the lipids were re-suspended in the starting reagent for Infinity (ThermoElectron, Waltham, MA) triglyceride reagent and triglyceride levels detected using the colorometric detection according to the manufacturer's specifications. Lipid levels are quantified using a standard curve of known triglyceride run in parallel.

### Heart rate

Heart rate was measured according to the methods laid out in (29). We anesthetized flies with 1 mL of FlyNap (Carolina Biological Supply) for 10 min (30). FlyNap does not affect heart function (31). Flies were attached to a slide with the dorsal side up by affixing their extended wings using double-stick tape. Flies were lit from below to see the heart contraction under 200X magnification. Contractions were counted over a 15 s period. The average of 5 separate observations per fly were used. There was a 15 s interval in between observation periods. The experimenter was blinded to genotype and counted the beats per 15 s in the fly.

### Resting metabolic rate

Resting metabolic rates were determined using a Sable Systems International CA-10 (Las Vegas, NV USA). Virgin female flies per genotype were collected using light CO_2_ anesthesia and put into vial until use. Metabolic rates were measured by using groups of 30, 5–7 day old flies at rest within flow-through respirometry chambers. Dry, CO_2_-free air was passed through the 10-ml glass respirometry chambers at 50 mL/min and then dried again and passed through a Li-Cor 6251 carbon dioxide analyzer (Lincoln, NE). Metabolic rates were measured for 5 min using constant respiromentry. The metabolic rate was the stable rate established. Within each run, seven experimental chambers containing flies were sampled in a sequential fashion by using a computer-controlled valve system. Genotypes were assigned randomly to the chambers for each run. One additional chamber was kept empty and sampled before and after each experimental chamber to remove any baseline drift. Temperature was measured by using a thermocouple within the empty chamber. Analog signals from the flow meter, carbon dioxide analyzer, and thermocouple were converted to digital and recorded on a computer (Sable Systems). After CO_2_ levels were measured, the weight of the flies was measured on a microbalance to normalize levels to tissue weight.

### Immunolocalization

Tissue from the Mi{Mic} flies inserted in *pdgy* were dissected and fixed in 4% paraformaldehyde in PBS for 1 h. Tissues was washed, blocked with normal goat serum, and then incubated overnight in 1:100 anti-GFP primary antibody (R&D Biosystems, Minneapolis, MN) at +4°C. After washing in PBS-T, the samples were incubated overnight in 1:100 Alexa Fluor 488 secondary antibody (Thermo Fisher, Eugene, Oregon) overnight at +4°C, and then washed and mounted in Vectashield. Images were taken using an Olympus IX51 inverted microscope at 600X total magnification using a UPLFLN 60X NA 1.25 objective and an MPlanFLN 10X NA0.30. FITC (EX 482/35 506DM EM 536/40) filter were used (Brightline). Images were captured with a Hamamatsu ORCA285 CCD camera. Shutters, filters, and camera were controlled using SlideBook software (Intelligent Imaging Innovations, Denver, CO).

### Data analysis and statistics

Where not otherwise stated, groups were analyzed by Student's *t*-test, in the case of comparing 2 samples and an ANOVA analysis for more than 2 groups with a *post hoc* Student's *t*-test to determine which groups are different.

## Results

**Figure 1:**
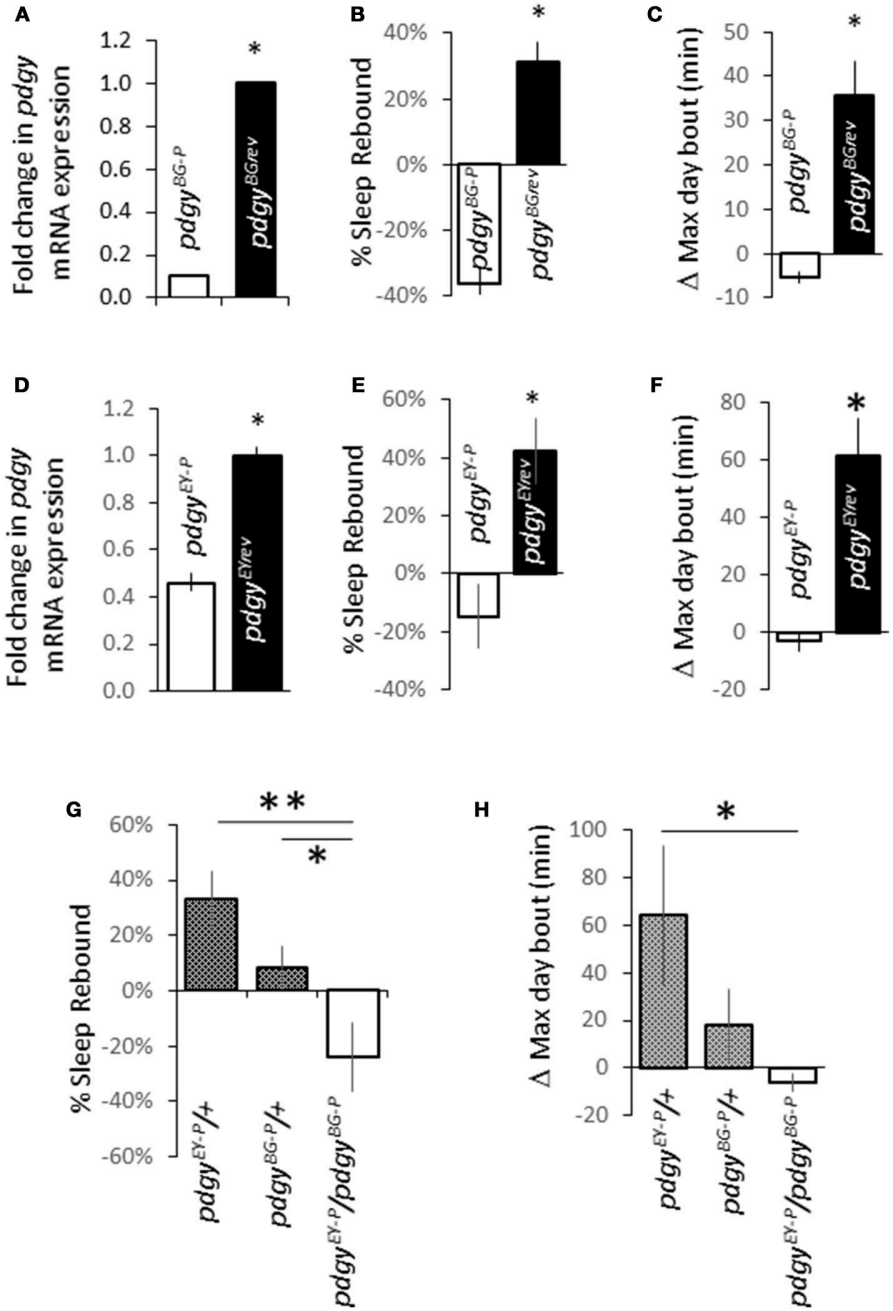
Disruption in *pdgy* expression alters the response to sleep deprivation. **(A)** Levels of *pdgy* mRNA are decreased in *pdgy*^*BG*02662^ (*pdgy*^*BG*−*P*^) flies compared to the background control, *pdgy*^*BGrev*^ (*n* = 3 groups of 5 pooled flies for each genotype, *p* < 0.05 by Students *t*-test). **(B)** The amount of sleep recaptured after sleep deprivation is significantly decreased when *pdgy* is disrupted (*n* = 49 *pdgy*^*BG*−*P*^ and *n* = 39 *pdgy*^*BGrev*^). **(C)** The individual change in the maximum day bout was increased in the *pdgy*^*BGrev*^ compared to the *pdgy*^*BG*−*P*^. **(D)** A second P-element near the *pudgy*^*BG*−*P*^ insertion and in a different background, *pdgy*^*EY*02124^ (*pdgy*^*EY*−*P*^) has decreased levels of *pdgy* RNA levels compared to the background controls, *pdgy*^*EYrev*^. **(E)** The sleep homeostatic response is muted in *pdgy*^*EY*−*P*^ (*n* = 40) compared to the background control *pdgy*^*EYrev*^ (*n* = 43). **(F)** The average increase in the maximum day bout is increased between the *pdgy*^*EY*−*P*^ and the *pdgy*^*EYrev*^. **(G,H)** To determine if there was something in the background responsible for the sleep phenotype in the background of the two strains of flies, we ran a complementation test. *pdgy*^*EY*−*P*^*/*+ (*n* = 16) and *pdgy*^*BG*−*P*^*/*+ (*n* = 14) showed a positive response to sleep deprivation while the *pdgy*^*EY*−*P*^*/pdgy*^*BG*−*P*^ (*n* = 13) heterozygote has a significantly lower sleep rebound **(G)** and maximum day bout on the day following sleep deprivation **(H)**. *p < 0.05, **p < 0.01.

We identified 2 independent P-element insertions within or near the first exon of the *pdgy* gene that could disrupt transcription or function, *pdgy*^*BG*02662^ (*pdgy*^*BG*−*P*^) and *pdgy*^*EY*02124^ (*pdgy*^*EY*−*P*^). We chose these two particular P-elements because they were created in two different backgrounds and were unlikely to have common mutations that might result in aberrant phenotypes (20). We first measured the RNA levels in the P-element compared to the revertant control. The revertants, *pdgy*^*BG*02662*rev*^ (*pdgy*^*BGrev*^) and *pdgy*^*EY*02124*rev*^ (*pdgy*^*EYrev*^) were generated by precisely excising the P-element to restore function in the same background as the P-element mutant. Both the *pdgy*^*BG*−*P*^ (Figure 1A) and the *pdgy*^*EY*−*P*^ (Figure 1D) had lower *pdgy* transcript levels compared to the revertants. To determine if *pdgy* impacts the response to sleep deprivation, we deprived flies of sleep for the 12 h primary sleep period from ZT13-ZT0. After sleep deprivation, both *pdgy* P-element alleles did not exhibit a sleep rebound after losing a full night's sleep. The background controls, *pdgy*^*BG*2*rev*^ (Figure 1B) and *pdgy*^*EYrev*^ (Figure 1E), had a large compensatory sleep rebound that were in the range of normal (23). Both the *pdgy*^*BGrev*^ (Figure 1C) and the *pdgy*^*EYrev*^ (Figure 1F) had increased average maximum day bout from ZT0-ZT12, an indicator of increased consolidation of sleep (32). The metric is compared the value from the baseline day before sleep deprivation to the same period the day after sleep deprivation (the 12 h post sleep deprivation). The increase in the maximum day bout indicates greater consolidation of sleep in the post-deprivation period, which is a property of the homeostatic response to sleep deprivation. Thus, the P-element mutations have an impaired ability to carry out a sleep rebound.

We then determined if the 2 P-elements or backgrounds complemented one another to rescue the phenotype. Heterozygous expression of each of the P-elements exhibited a rebound whereas the *pdgy*^*BG*−*P*^/*pdgy*^*EY*−*P*^ heterozygous animal exhibited a significantly negative rebound (Figure 1G). The change in the maximum day bout was increased between the *pdgy*^*EY*02124^*/*+ and the *pdgy*^*BG*02662^/*pdgy*^*EY*02124^ but not a statistical difference between the *pdgy*^*BG*02662^*/*+, though the outcross showed a positive change and the heterozygote had a decrease in the max day bout (Figure 1H). Thus the sleep rebound phenotype is due to the *pdgy* locus and genetically supports the conclusion that both alleles have decreased levels of *pdgy* that do not compensate for each other.

**Figure 2:**
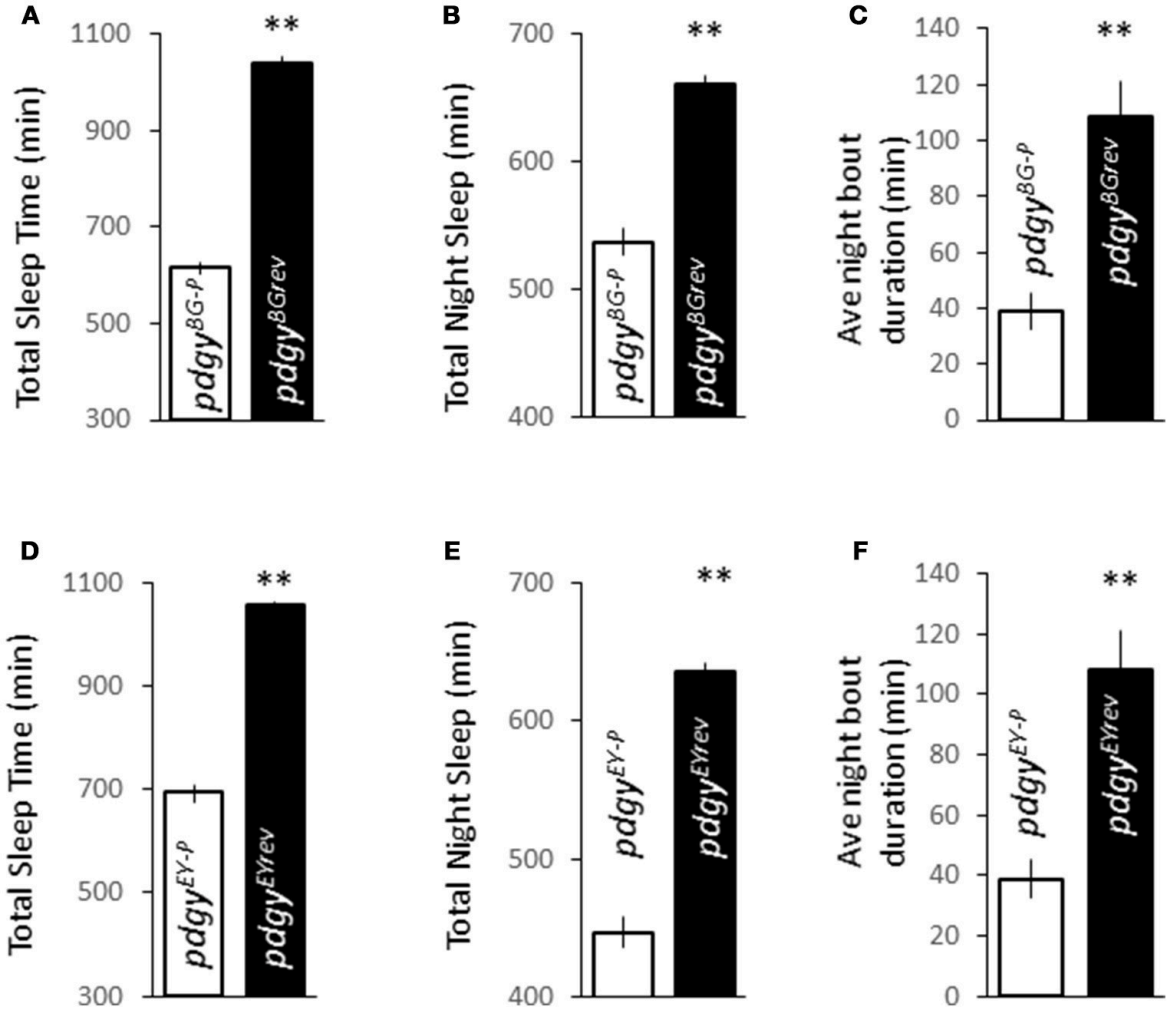
Disruption in *pdgy* expression alters baseline sleep characteristics. **(A–C)** Flies with decreased pdgy expression, *pdgy*^*BG*−*P*^ (*n* = 84), show reductions in sleep with sleep times over 24 h sleep duration throughout the night **(B)**, **(A)** and sleep consolidation as measured by average night bout duration **(C)** compared to background control *pdgy*^*BGrev*^ (*n* = 63). **(D–F)** Total sleep time **(D)** nighttime sleep duration **(E)**, and night and consolidation **(F)** are decreased in flies with decreased *pdgy* (*pdgy*^*EY*−*P*^) (*n* = 89) compared to background controls (*pdgy*^*EYrev*^) in a second background (*n* = 68). **p < 0.01 by Student's *t*-test.

In the course of the sleep deprivation experiments, we observed that the flies with the transposon insertion sleep considerably less. In fact they had a decreased overall duration (Figures 2A,D), with a striking decreased in night duration (Figures 2B,E), the fly's most consolidated sleep period. In addition, these flies were significantly more fragmented than their background controls (Figures 2C,F). Unlike mutations in other lipid metabolism genes in *Drosophila, pdgy* reduces baseline duration and consolidation in the primary sleep period of both the *pdgy*^*BG*−*P*^ and *pdgy*^*EY*−*P*^ mutants.

**Figure 3:**
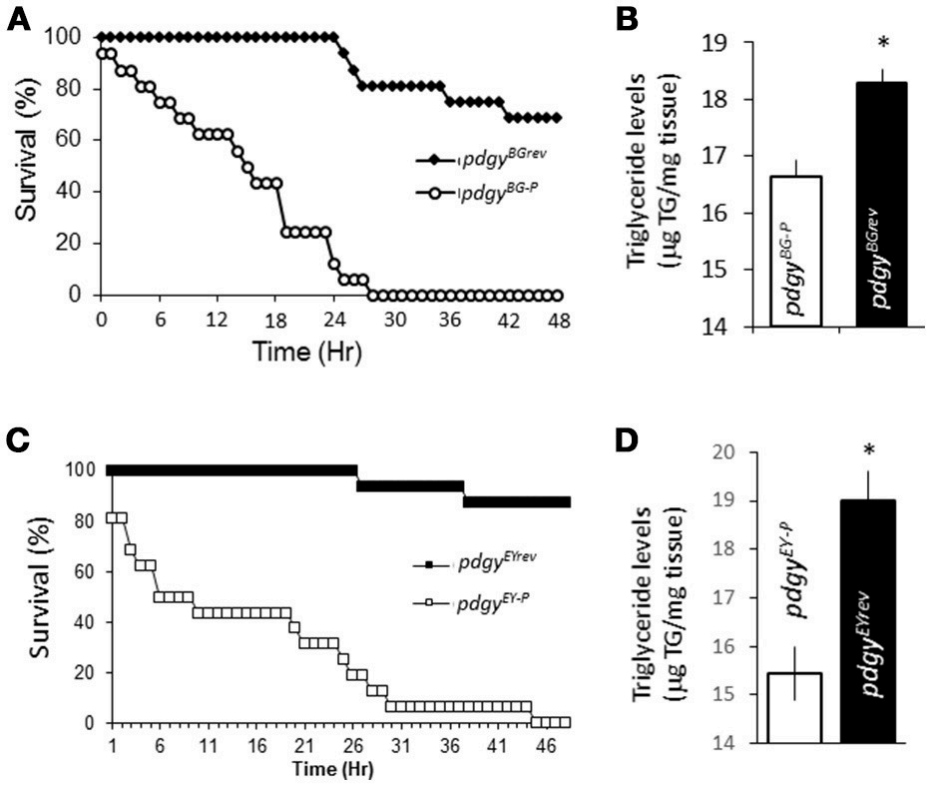
*pdgy* mutants display metabolic phenotypes. **(A)** Under starvation conditions, the *pdgy*^*BG*−*P*^ displayed decreased survival compared to background controls, *pdgy*^*BGrev*^. **(B)** *pdgy*^*BG*−*P*^ had decreased lipid stores compared to background controls. **(C,D)** Starvation phenotypes compared to its background control (*pdgy*^*EYrev*^) as the *pdgy*^*BG*−*P*^ **(D)** and *pdgy*^*EY*−*P*^ shows a similar triglyceride phenotypes. *p < 0.05.

We then confirmed whether our *pdgy* hypomorphs showed metabolic phenotypes compared to the background controls. We started by determining the fly's starvation sensitivity. After 48 h of starvation, ~75% of the *pdgy*^*BGrev*^ were still alive while the *pdgy*^*BG*−*P*^ mutant flies had completely died after approximately 27 h (Figure 3A). The same relationship was observed with the *pdgy*^*EYrev*^and *pdgy*^*EY*−*P*^ flies (Figure 3C). These results suggest that there is a decreased levels of energy stores or a decreased ability to mobilize those resources under starvation conditions. We directly measured the triglyceride levels. In our hands, the *pdgy*^*BG*−*P*^ had decreased triglyceride levels compared to the *pdgy*^*BGrev*^ (Figure 3B) and the relationship was similar between the EY mutant and background control as well (Figure 3D). Thus, it appears that both the *pdgy*^*BG*−*P*^ and *pdgy*^*EY*−*P*^ had decreased triglyceride levels compared to the revertants and less available resources to mobilize. Though these results do not preclude an inability to properly mobilize or utilize liberated free fatty acids.

**Figure 4:**
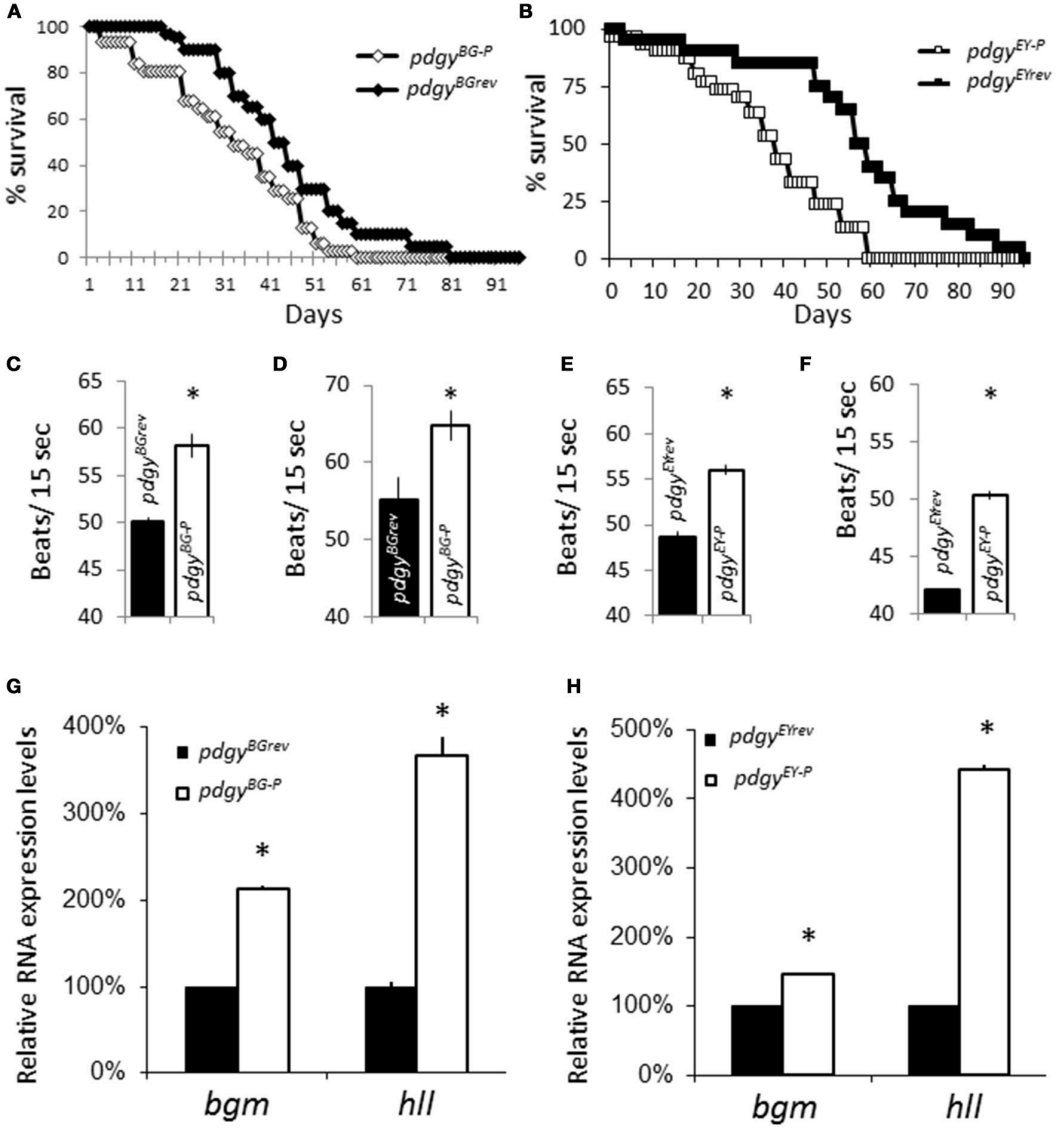
Consequences of the *pdgy* mutants. **(A)** Lifespan measure for the *pdgy*^*BG*−*P*^ (*n* = 30) compared to the revertant control, *pdgy*^*BGrev*^ (*n* = 30). **(B)** Lifespan measure for the *pdgy*^*EY*−*P*^ (*n* = 30) compared to the revertant control, *pdgy*^*EYrev*^ (*n* = 30). *pdgy*^*BG*−*P*^ has increased heart rates compared to background controls at **(C)** 9–11 d.o. and **(D)** 22–25 d.o. A similar phenotype is observed in *pdgy*^*EY*−*P*^ compared to background controls at **(E)** 9–11 d.o. and **(F)** 22–25 d.o. **(G)** Levels of the ACSs *bubblegum* (*bgm*) and *heimdall* (*hll*) are elevated in the *pdgy* hypomorph (*pdgy*^*BG*−*P*^ in **G**) and (*pdgy*^*EY*−*P*^ in **H**) compared to the revertant control. *p < 0.01.

Both decreased sleep durations and fragmented sleep have been associated with adverse consequences related to inadequate sleep. We assessed whether lifespan was decreased in the mutants. *pdgy*^*BG*−*P*^ had a 50% survival at ~33 days and a final lifespan to 59 days while the *pdgy*^*BGrev*^ had a 50% survival at 42 days and final survival at 81 days (Figure 4A). A similar phenotype was observed in *pdgy*^*EY*−*P*^ as the 50% survival was at 38 days and the genotype survived till 59 days while *pdgy*^*EYrev*^ had a 50% survival at day 50 and a final survival for 95 days (Figure 4B). These results are consistent with previous results from inadequate sleep.

Another consequence of poor sleep is that there are increases in heart rate. We evaluated heart rate in the flies. Heart rate was elevated in both sets of mutants compared to their revertant controls at 9-11 days old (Figures 4C,E). We were interested if the phenotype would progress as the flies aged because the consequences of low sleep duration and sleep fragmentation would continue to build up. In flies that were 22–25 days old, both sets of mutants still had an elevated heart rate, but the phenotype did not appear to get worse with increased time (Figure 4D,F). The increased heart rate is consistent with phenotypes associated with inadequate sleep. We reasoned that the similar phenotypes might have similar compensatory responses to in other ACSs. Both hypomorphic lines showed increased transcript levels of *bgm* and *hll* compared to revertant controls, indicating that there are modifications in lipid processing enzymes in response to decreased levels of *pdgy* (Figures 4G,H).

**Figure 5:**
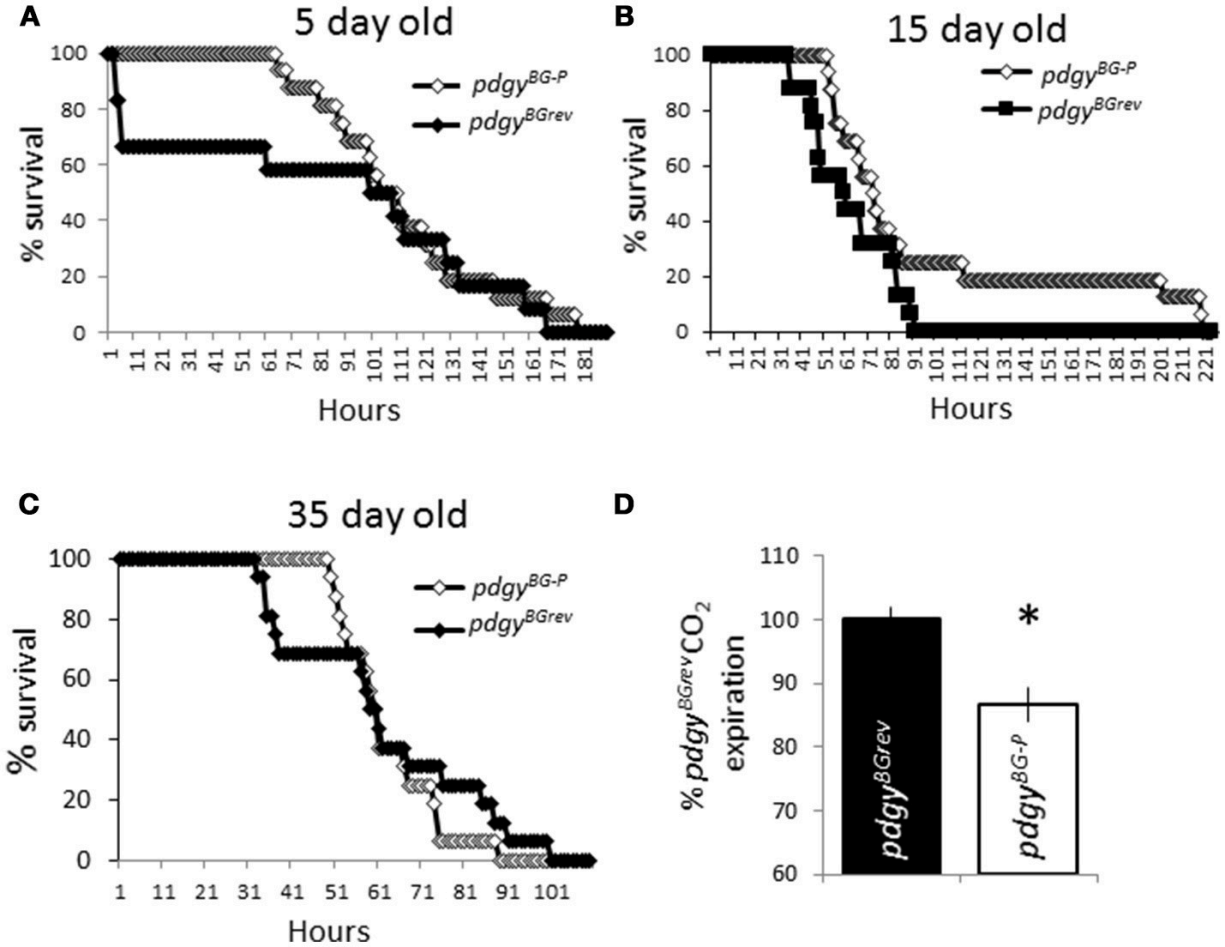
*Pdgy* mutants are not generally unfit flies. *pdgy*^*BG*−*P*^ and *pdgy*^*BGrev*^ were put on peroxide in their food different ages, 5 d.o. **(A)**, 15 d.o. **(B)**, and 35 d.o. **(C)**. *n* = 16 for each condition. **(D)** We measured levels of CO_2_ expired as an indicator of respiration. Levels of CO_2_ are displayed as percent of control. *p < 0.05.

Since the flies displayed a decreased lifespan, we wanted to determine if the flies were generally weaker than the background flies. When the flies were challenged with hydrogen peroxide, flies with the P-element integrated into the genome were able to tolerate the infusion of a compound known to increase cellular stress throughout the animal (33). We evaluated young mutant flies and the background revertants (Figure 5A). To our surprise, the P-element mutants appeared to be more resilient to the infusion of peroxide compared to the background revertants at 5 days old. It is possible that with age, the P-elements would have shown an increasing susceptibility with age. We then tested middle age and older flies as well. In each case, the P-element flies were more resilient to peroxide treatment than the background controls (Figures 5B,C). Interestingly, the peroxide had the intended result as the number of hours that the population survived on peroxide decreased with age but the relationship between the genotypes was not changed. These results indicate that the P-element flies are not inherently weaker than the background controls.

We evaluated whether the resting metabolic rate was different between the *pdgy* mutants and background controls (Figure 5D). Interestingly, the metabolic rate was lower in the *pdgy* mutants, perhaps reflecting the decreased ability to appropriately process fatty acids. In addition, these data suggest that the respiration rate is not higher in the mutant flies.

**Figure 6:**
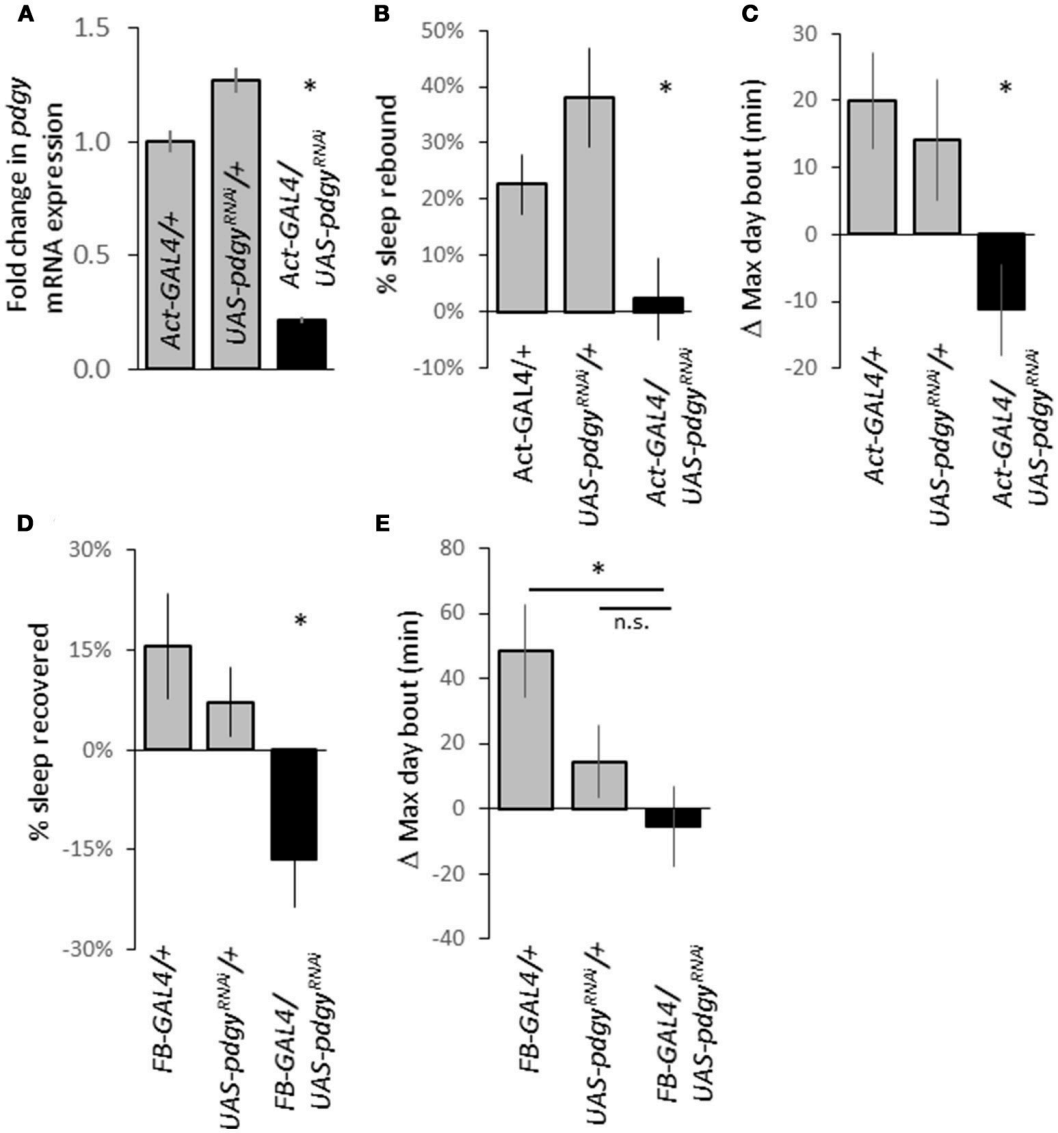
*Pdgy* knockdown results in a decreased rebound. **(A)** We confirmed knockdown using real-time qPCR as RNA levels were decreased in *Act-GAL4*>*UAS-pdgy*^*RNAi*^ compared to background controls. **(B)** Ubiquitous *pdgy* RNAi knock down (*Act-GAL4*>*UAS-pdgy*^*RNAi*^) reduced the response to sleep deprivation compared to the background controls, *Act-GAL4/*+ and *UAS-pdgy*^*RNAi*^/+. N = Act 50, UAS = 38, Act/UAS = 40). **(C)** An increase in the maximum day bout was suppressed when pdgy was knocked down ubiquitiously. **(D)** Knockdown in the fat body (*FB-GAL4*>*UAS-pdgy*^*RNAi*^) also decreased the response to sleep deprivation compared to background controls FB-GAL4/+ and *UAS-pdgy*^*RNAi*^/+. **(E)** The change in the maximum day bout in *FB-GAL4*>*UAS-pdgy*^*RNAi*^ compared to background controls. For **(D,E)**, *N* = *FB-GAL4/*+ = 29, *UAS-pdgy*^*RNAi*^/+ = 53, *FB-GAL4/UAS-pdgy*^*RNAi*^ = 32). *p < 0.05.

The P-element mutations suggested a decreased level of *pdgy* was responsible for the decreased duration and increased sleep fragmentation. We used an independent knockdown technique guided by the bipartite GAL4-UAS system to decrease levels of *pdgy* (34). We knocked down *pdgy* throughout the animal using the ubiquitous driver *Actin-GAL4* (*Act-GAL4*). We confirmed knockdown as levels of *pdgy* transcript were about 20% in the *Act-GAL4*>*UAS-pdgy*^*RNAi*^ of what they were in the background controls (Figure 6A). When the animal was sleep deprived, the control lines, *Act-GAL4/*+ and the *UAS-pdgy*^*RNAi*^*/*+ flies showed a significantly higher rebound in sleep rebound compared to the experimental line, *Act-GAL4*>*UAS-pdgy*^*RNAi*^ (Figure 6B). The maximum day bout was lower in the knockdown flies compared to both controls (Figure 6C). We then wanted to determine if a more localized knockdown in the fat body would alter the rebound using the *Fat body-GAL4* (*FB-GAL4*), an enhancer trap that has been shown to express in the adult fly fat body (35). When *pdgy* is reduced using *FB-GAL4*, the rebound to sleep deprivation was again reduced below the background lines, indicating that the knockdown of *pdgy* in the fat bodies may contributes to the sleep rebound phenotype (Figure 6D). When max day bout was assessed, it was lower in the knockdown flies compared to the *FB-GAL4/*+ background flies but did not reach significance in compared to *UAS-pdgy*^*RNAi*^*/*+ flies (Figure 6E). Though, the *UAS-pdgy*^*RNAi*^*/*+ flies showed an increase while the knockdown flies showed a decrease in max day bout, which is consistent with how the sleep rebound was expressed. With both drivers, we did not observe the baseline sleep phenotype, possibly because the knockdown was not strong enough or in the appropriate cells.

**Figure 7:**
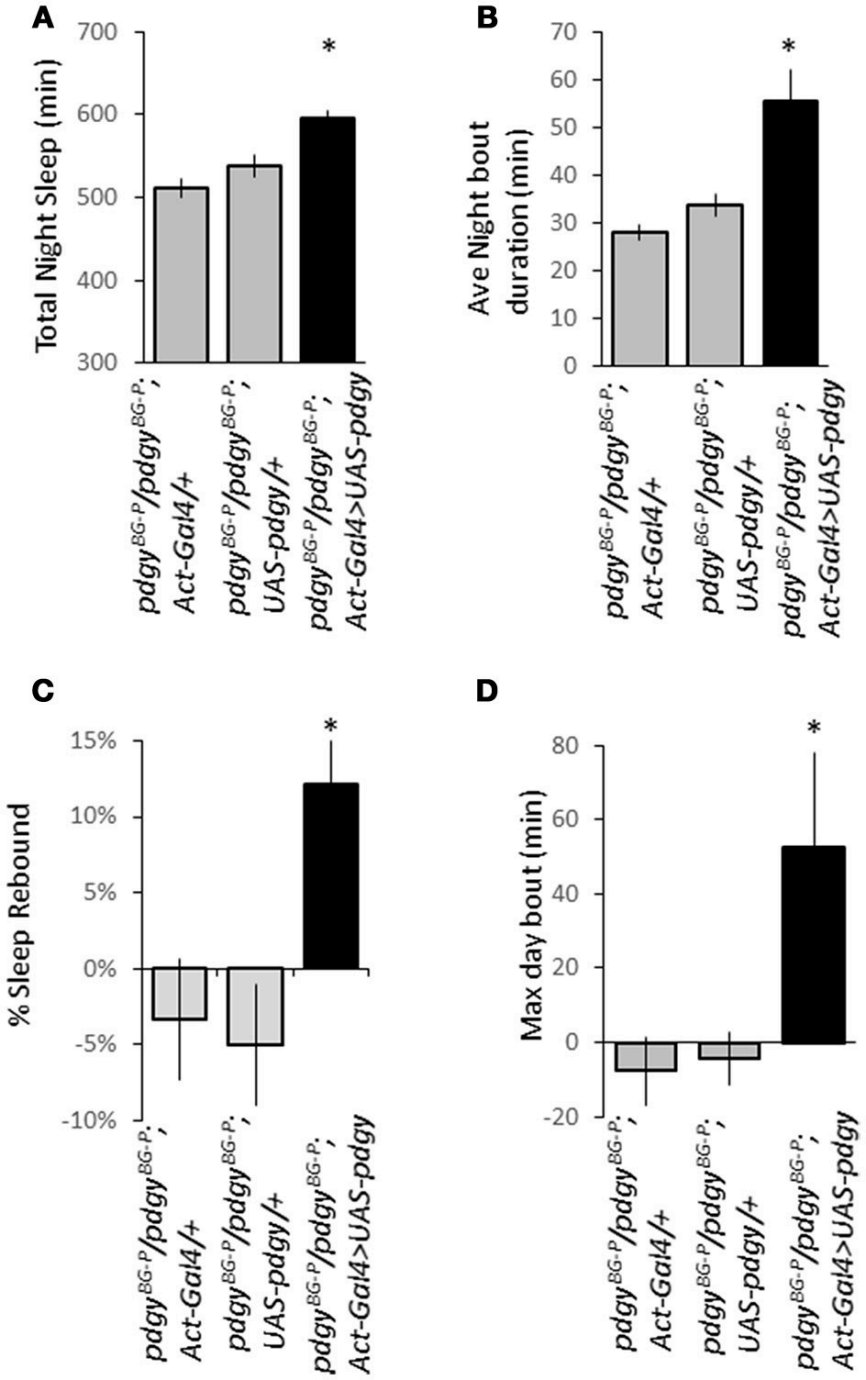
Rescue of pdgy in the *pdgy*^*BG*−*P*^ background restores sleep homeostasis, duration, and consolidation. Re-introduction of the wild-type version of *pdgy* (dark bars) restores the duration of night time sleep **(A)** and increases the average night bout duration as a measure of sleep consolidation **(B)** compared to the driver (*Act-GAL4/*+) and the responder (*UAS-pdgy*) both in gray. (For both **A,B**, *n* = 63 for Act-GAL4/+, 64 for UAS-pdgy/+, and 61 for Act-GAL4>UAS-pdgy). **(C)** Rescue also restored a significant rebound after sleep deprivation. **(D)** The maximum day bout is increased on the first recovery day after sleep deprivation. For **(C,D)**, *n* = 29 for Act-GAL4/+, 26 for UAS-pdgy/+, and 30 for Act-GAL4>UAS-pdgy. *p < 0.05.

As a final confirmation that *pdgy* was a critical part of the sleep rebound, we employed a rescue strategy in which the wild-type *pdgy* transcript was expressed ubiquitously in an otherwise *pdgy* hypomorphic animal. We put both *Act-GAL4* and *UAS-pdgy*^*wt*^, generously provided by Aurelio Teleman (18), into the *pdgy*^*BG*−*P*^ background. The rescue flies exhibited increase night sleep (Figure 7A) and increased night time consolidation (Figure 7B) compared to flies that did not carry the rescue construct. In addition, rescue flies had a homeostatic response to sleep deprivation whereas mutant flies did not (Figures 7C,D). Thus, these data indicate that rescuing *pdgy* restores more wild-type sleep traits.

**Figure 8:**
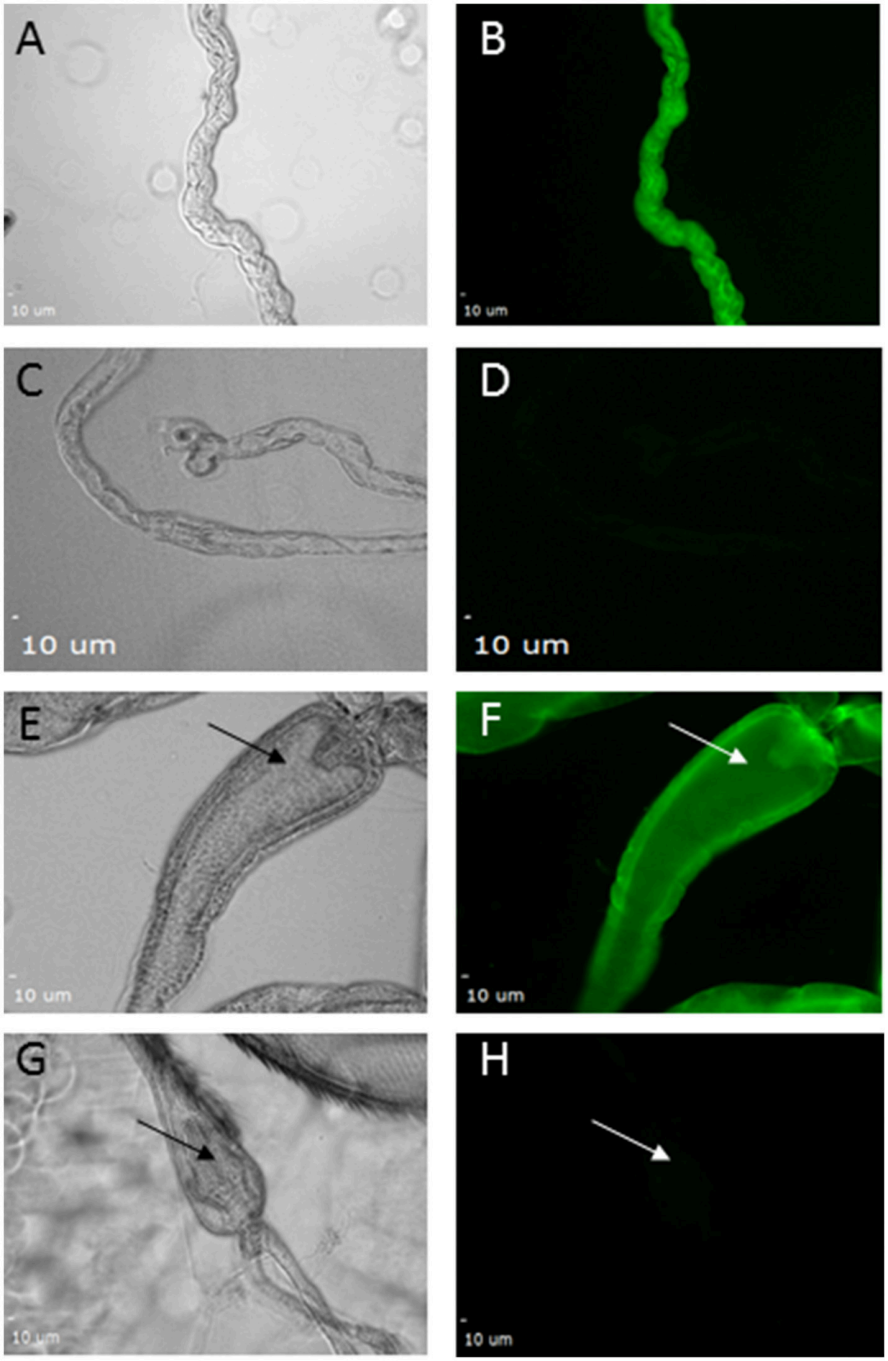
Expression of pdgy was detected in the gut and Malpighian tubules using an enhancer trap line. GFP expression was detected in the Malpighian tubules **(A–D)** and in the gut **(E–H)**. Bright field images show Malpighian tubules **(A,C)**. Fluorescence expression was detected in **(B)** when the secondary antibody was excited by wavelengths in the green wavelengths as opposed to a control **(D)** with a secondary antibody not excited or emitting in this same wavelength. GFP expression was also expressed in the gut. Shown here is the recognizable portion of the gut, the cardia. Expression was seen in **(F)** but not in **(H)** with the same conditions as above. Bright field images showing the field **(E,G)**. Arrows point to the cardia in each image.

Since knockdown using *FB-GAL4* did not phenocopy the *pdgy* hypomorphs, we wanted to determine if there was *pdgy* expression elsewhere in the animal. We took advantage of a set of flies in which an EGFP tag has been integrated into the *pdgy* gene through random integration. This allows us to visualize where the *pdgy* gene is expressed endogenously. This technique comes with inherent caveats, but it has also revealed novel expression patterns and generated interesting hypotheses when an antibody is unavailable, as it is with *pdgy*. Using this technique, we observed faint *pdgy* expression in the fat bodies. We did observe expression in the Malpighian tubules (Figures 8A–D) as well as throughout the gut with particular localization in the cardia of the gut (Figures 8E–F). In addition, we observed expression in the Malpighian tubules (Figure 8B). Thus, the expression of *pdgy* may be more widespread in the fly and it is possible these organs could play a role in the response to sleep deprivation.

## Discussion

Lipid metabolism has become a critical regulator of sleep and the response to sleep deprivation. The role of metabolism is taking a more prominent role in governing sleep regulation and the response to sleep deprivation (1, 36–38). During sleep there is very small drop in energy expenditure (39), suggesting that there is a substantial energy requirement, even during sleep. In microarray studies, lipid metabolism genes have elevated transcription in the brain in both mammals and flies (11, 40). In humans, the brain is active during sleep, which will consume energy, especially during rapid eye movement (REM) sleep (41, 42). In addition, energy consumption continues throughout the body during sleep, resulting in only a modest decrease in energy consumption compared to waking (39). Energy may play a critical role because both waking activities and the restorative activities of sleep require energy to carry out their functions (1). Thus, molecules and enzymes that control energy metabolism may regulate sleep and wake cycles and disruptions or changes in metabolic handling and the signals, such as endocrine signals, may have an impact on sleep behavior.

We have been evaluating the role that lipid metabolism plays in regulating the response to sleep deprivation (3). Through a microarray that compared waking that induced damage to the organism compared to waking that does not result in performance and health decrements, we identified several lipid metabolism genes, including the ACSs *bgm* and *heimdall* (*hll*) (4). One lipid metabolism gene, the ACS *pdgy*, was not identified, whereas *pdgy* (identified as *CG9009*) had been identified as a sleep regulatory gene (11). It remains unclear if *pdgy* plays a role in sleep regulation or the response to sleep deprivation.

Here, we have demonstrated that the acyl-CoA synthetase, *pdgy*, plays a critical role in the response to sleep deprivation. This is consistent with the role that other lipid metabolism genes in the fly (3, 4). For example, mutations in ACSs, such as *bgm* and *hll*, result in decreases in the sleep rebound. Knockdown of *hll* also has a lipid phenotype as lipid levels were lower but retained the distribution in mutant flies as measured by lipidomics (4). In contrast with other ACSs, we also demonstrated that *pdgy* also reduced the night sleep duration and increased fragmentation, both of which decrease the restorative ability of sleep. *Pdgy* (or *CG9009* as it was previously designated) had been identified as a gene that increased its transcription levels with sleep deprivation. (11). Thus, we provide strong evidence that *pdgy* is involved in sleep regulation and the response to sleep deprivation.

**Figure 9:**
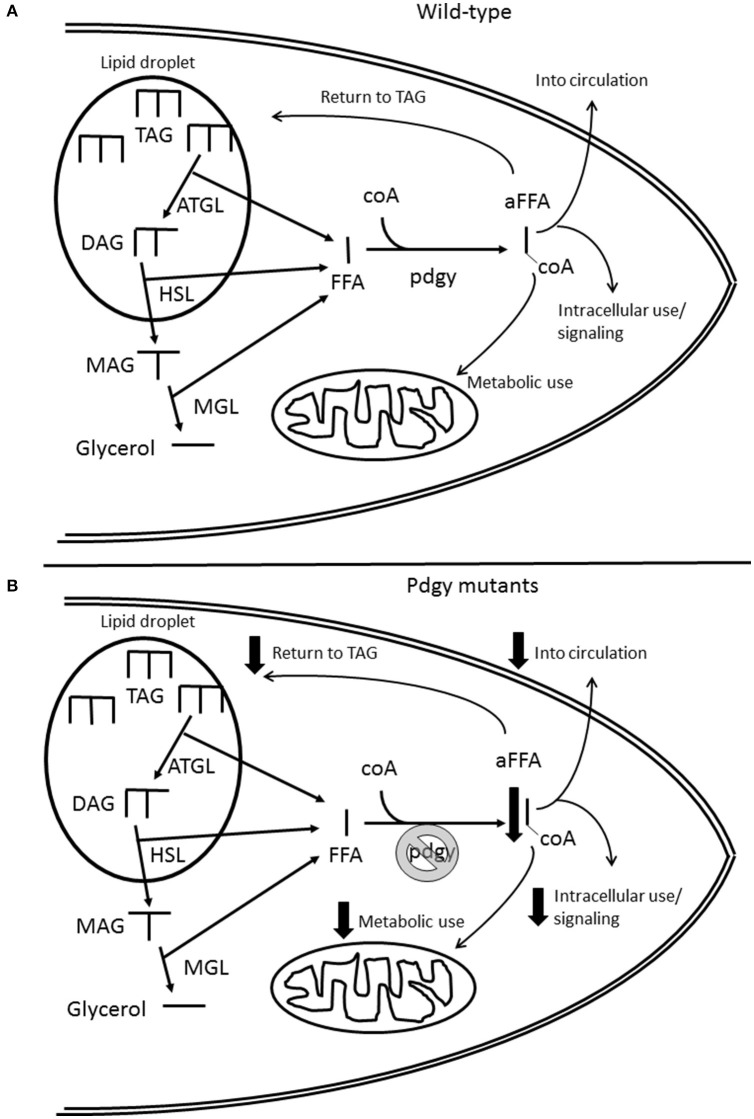
Schematic of the role of pdgy in cellular lipid metabolism. **(A)** Under normal conditions, the adipose triglyceride lipase (ATGL) releases free fatty acids (FFA) and diacylglycerides (DAG) from the lipid droplet. Another FFA is released from DAG by hormone sensitive lipase (HSL) and another FFA is released from the Monacylglyceride (MAG) by monoglyceride lipase (MGL) leaving glycerol. Pdgy then adds a coA molecule to the FFA to activate it (aFFA). The aFFA then is used for metabolic, may be reinserted into the lipid droplet, cell signaling or protein post-translational modifications, or distribution to other tissues through the circulation. **(B)** The absence or reduction of pdgy would result in less FFA coverted to aFFA. This mutation would potentially reduce the FFA available to carry out necessary functions within the cell and throughout the organism. Another consequence may be the increase in the levels of FFA within the cell.

*Pdgy* has been identified as an ACS (21), metabolic enzymes that activate fatty acids. These fatty acids can act as signaling molecules, fulfill cellular functions or transferred into the mitochondria for oxidative phosphorylation to generate energy (Figure 9). In that study, the authors hypothesized that the *pdgy*^*BG*−*P*^ had a difficulty utilizing stored triglycerides. The results presented here agree with those conclusions. In the starvation experiments, *pdgy*^*BG*−*P*^ and *pdgy*^*EY*−*P*^ died in about 24 h without food, whereas the majority of the revertants were still alive after 48 h of starvation. In addition, we found a triglyceride phenotype, suggesting that *pdgy* is involved in lipid metabolism. Where the manuscripts do not match is in the precise metabolic outcomes. In Xu et al., the *pdgy*^*BG*−*P*^ had elevated triglycerides compared to the controls, and in a starvation assay, the *pdgy*^*BG*−*P*^ flies exhibited increased starvation resistance. In our study, we also examined another P-element mutation *pdgy*^*EY*−*P*^ also show a metabolic phenotype similar to the *pdgy*^*BG*−*P*^. Since these mutations were generated in two different backgrounds, the BG lines were made in an isogenized line while the EY lines were made in a different background that were more conducive to P-element integration (20), these two lines do not carry the same background mutations and gene expression levels. In addition, when the two P-element lines were crossed to each other, there was no complementation, again suggesting that pdgy was responsible for the sleep phenotype. Therefore, the phenotypes observed in the *pdgy*^*BG*−*P*^ and *pdgy*^*EY*−*P*^ are likely due to the *pdgy* gene and not second-site mutations for 2 different lines.

On its face these results may seem incompatible, but the relative differences may be due to how each of the mutants were generated. In Xu et al., they outcrossed the BG mutant five times to a white mutant, which in theory exchanges the background around the P-element for the background on the white-eyed flies. The white-eyed mutant is then the comparator genotype. This method standardizes the background and make it easier to compare between different mutants. The white-eyed mutant background is not a preferable background, just a standardized background with all of the mutations and differences that occur in the white background. Our approach was to precisely excise the P-element to restore *pdgy* transcript levels in the same background that the original mutation was in. This is a common method to restore function in flies. Therefore, our comparator group is different than their comparator group and triglyceride levels would be relatively different. The metabolic system is complex and may have complex interactions. Therefore, in the Xu et al. experiments, the background may allow enough of the lipids to be processed to permit starvation resistance whereas in the *pdgy* revertant background may not allow lipid utilization that supports survival under starvation conditions.

These results highlight how the difference in genetic context can lead to serious consequences or not so serious consequences. One example of where this has occurred is with the CLOCK mutant mouse. In one case, the impact of the mutation was increased weight gain, lipids dysregulation and cholesterol possibly due to the lack of circadian rhythms (43). Yet when that same mutation was moved into a second background, the opposite phenotype was observed (44). In both cases, circadian rhythms were disrupted, but the consequences were different in the different circumstances. These results may have a clinical implication as shift workers, who often have misaligned circadian rhythms, have an increased likelihood to be obese (45). Interestingly, understanding the differences between the two backgrounds may provide insights into possible treatment options to prevent lipid dysregulation in people with circadian disruptions.

We have gone on to demonstrate using other genetic methods that *pdgy* plays a role in the response to sleep deprivation. The sleep deprivation phenotype was replicated using both knockdown and rescue indicating that *pdgy* is necessary and sufficient to play a role in this process. The baseline sleep phenotype was replicated in the rescue experiments. We hypothesize that the knockdown was not strong enough in the appropriate cells to impact the sleep duration and fragmentation phenotypes, but that presents an opportunity to identify cells responsible for these phenotypes. Moreover, the phenotypes were observed in 2 different P-element lines with 2 different backgrounds. In sum, the genetics strongly implicates the *pdgy* in sleep regulatory pathways.

In the fly disruptions in the metabolic system have been shown to alter the response to sleep deprivation. Presumably, the energy demand is created to carry out the restorative aspects of sleep. At a cellular level, the restorative role that sleep plays will require energy to convert and/or dispose of the damage generated during waking activities. Under most normal circumstances that energy is limited (1). Therefore during sleep deprivation the body cannot satisfy the energy demands of both restoration and waking functions and must leave some things undone. Consistent with this hypothesis, levels of circulating free fatty acids are increased with sleep deprivation, consistent with an increase in available energy with sleep deprivation (10). These incomplete functions may underlie the deleterious consequences of waking. Mutations that decrease triglyceride lipase activity inhibit the response to sleep deprivation. Moreover, a mutation in the gene *Lipid storage droplet-2* (*LSD-2*), results in increased triglyceride lipase activity and a blunted sleep rebound (3). Interestingly, starved flies (both the *cycle*^01^ mutant and the *w*^1118^ line) and *LSD-2* mutant flies were able to learn after the same amount of sleep loss as sleep deprived flies that showed learning impairments. What these two conditions have in common is that they both have elevated levels of lipolysis that could sustain increased energy needs. In other animals there are temporary periods in which sleep is disrupted, but function is maintained. Female orcas and dolphins that have just given birth remain awake with their calves for approximately a month without a subsequent increase in sleep to compensate (46). White-crowned sparrows retain cognitive function during the migratory season, despite substantial sleep deprivation (47). Cognitive function is not retained if the sleep loss is outside of the migratory period. Sandpipers are able to maintain reproductive fitness in the face of sleep deprivation (48). This possibility of increased lipolysis is consistent with all of the natural conditions in which sleep deprivation is not accompanied by impairments from sleep loss. These data support the need for lipid metabolism to support sleep and the response to sleep deprivation though the effects are a complex process and may depend on the genetic and environmental context (49). The *pdgy* sleep phenotypes observed here are consistent with other lipid metabolism mutants that do not permit a homeostatic response to sleep deprivation.

We evaluated potential consequences of short sleep duration and increased fragmentation sleep in the P-elements. In other situations, short sleep increases cardiovascular effects, including heart rate potentially due to increases in the sympathetic system (50, 51). Moreover, short sleep duration has been associated with shorter lifespan in flies and humans (52–54). The phenotypes observed, elevate heart rate and decreased longevity, were consistent with the consequences of inadequate sleep. These occurred in both P-element mutants, but these flies were not more sensitive to stresses as measured by exposure to hydrogen peroxide. These consequences were not observed in either the RNAi knockdown experiment or the rescue experiments in the *pdgy*^*BG*−*P*^ background. The GAL4/+ in the BG background showed the decreased lifespan, but the UAS/+ did not shown the decrements in lifespan on the same timeline as the rescue experimental line. This despite the fact that sleep duration was decreased and sleep was fragmented in the background lines, but was normal in the rescue line. This result argues against that sleep loss and fragmentation result in a reduced lifespan phenotype. Alternatively, the rescue expression of *pdgy* may provide protection under these circumstance. Another possibility is that in the P-element background, the lack of proper fatty acid utilization results in the observed phenotypes.

We used an enhancer trap line to localize pdgy within the fly. Enhancer traps integrate an exogenous piece of DNA into the animal's DNA. Once present, expression of the GFP falls under the control of local enhancer and repressors to guide expression. For pdgy, the MI{MIC} line is integrated within the pdgy gene and thus likely reflects endogenous expression. Our results demonstrated strong expression in the cardia and throughout the gut as well as expression in the Malpighian tubules, the kidney-like organ in the fly. We didn't see strong expression in the adipose tissue, though we did not test it under starvation conditions where there is likely a large upregulation of pdgy in the fat bodies (18). In mammals, ACSs have been found in the intestinal epithelial cells (55), supporting the interpretation that MI{MIC} expression mimics endogenous expression. Another possibility is that the MI{MIC} expression responds to only part of the enhancer control. As we did not see complete phenocopy between the RNAi knockdown in the fat body and the P-element hypomorphs. Therefore it is possible that this expression pattern may contribute to sleep regulation.

The fact that lipid metabolic enzyme *pdgy* can alter sleep regulation has a two broader implications. First, this is further evidence that lipid metabolism plays a critical role in sleep regulation under baseline conditions and after sleep deprivation. Energy management appears to be an obvious route to impact sleep regulation, but this does not rule out a contribution by lipid signaling. Secondly at the physiological level, endocrine signals change the expression of metabolic enzymes. For example, both growth hormone and insulin alter the transcription for lipogenesis (56). Given that there are numerous ACSs that likely have specificity for chain lengths of fatty acids, not all of these changes would have the same impact on sleep regulation as *pdgy* it will alter the cellular metabolic environment. In fact, starvation alters the metabolic environment and increases wakefulness (3, 57–59). Therefore, when the hormonal environment changes because of dietary conditions or because of disease, one of the consequences could be that the fatty acid mobilization and activation enzymes are changed and that those changes may impact sleep regulation and the response to sleep deprivation. In addition, inadequate sleep can alter the endocrine environment (37, 60–62). Thus, there is a direct connection between the endocrine system to sleep regulation through metabolic enzymes.

## Author contributions

MT designed experiments, ran experiments, analyzed data, and wrote the manuscript. NK, JL, CF, and TH all designed and performed experiments, and analyzed the subsequent data.

### Conflict of interest statement

The authors declare that the research was conducted in the absence of any commercial or financial relationships that could be construed as a potential conflict of interest.
